# Enhancing Higher-Order Cognitive Abilities Through AI-Powered Smart Assistants: Implications for Digital Entrepreneurship and Future Problem-Solving in Higher Education

**DOI:** 10.3390/jintelligence14080170

**Published:** 2026-08-01

**Authors:** Ahmed Sadek Abdelmagid, Naif Mohammed Yahya Jabli, Adel Ibrahim Qahmash

**Affiliations:** Department of Education and Learning, College of Education, King Khalid University, Abha P.O. Box 157, Saudi Arabia; njabli@kku.edu.sa (N.M.Y.J.); aqahmash@kku.edu.sa (A.I.Q.)

**Keywords:** artificial intelligence, AI-powered smart assistants, higher-oriented cognitive abilities, digital entrepreneurship, future-oriented problem solving, instructional design, higher education

## Abstract

Higher education is undergoing a rapid transformation driven by advancements in artificial intelligence (AI), creating new opportunities to support the development of higher-order cognitive skills through carefully designed learning environments. This study aimed to examine the effectiveness of an educational program that integrates AI-powered intelligent assistants into a structured e-learning environment to support the development of digital entrepreneurship and future-oriented problem-solving skills among graduate students. A quasi-experimental design was used with 82 graduate students, randomly assigned to two groups: an experimental group (*n* = 41) and a control group (*n* = 41). Both groups studied the same educational content and completed identical learning activities; however, the experimental group participated in a structured learning framework that included AI-powered intelligent assistants, guided learning activities, and instructor facilitation, while the control group completed the same activities without AI support. The educational program comprised five modules focusing on chatbot design, intelligent platform development, digital content production, educational data analysis, and future-oriented problem-solving. Learning outcomes were assessed using a Digital Entrepreneurship Product Rating Scale and a Future-Oriented Problem-Solving Scale, which measures skills in visualization, prediction, foresight, and planning. The results revealed statistically significant differences favoring the experimental group on both outcome scales, with large effect sizes. These findings suggest that integrating AI-powered intelligent assistants within a structured learning framework may support the development of digital entrepreneurship and future-oriented problem-solving skills among graduate students. This study contributes empirical evidence to the potential educational value of integrating AI-powered intelligent assistants with pedagogically designed learning activities to foster higher-level cognitive development in higher education.

## 1. Introduction

Recent advances in generative artificial intelligence have radically changed the role of digsital technologies in higher education. Rather than being limited to retrieving information or providing educational support, AI-powered intelligent assistants are increasingly being viewed as cognitive partners that augment human reasoning by supporting reasoning, organizing knowledge, providing adaptive feedback, and supporting cognitive regulation. This perspective is consistent with contemporary cognitive science, which views intelligence as a dynamic process that includes the construction and transfer of knowledge and adaptive problem solving, rather than simply the passive acquisition of information. As a result, when AI-powered intelligent assistants are integrated into pedagogically meaningful learning environments, they have the potential to enhance higher-order cognitive abilities, including analytical reasoning, decision-making, creativity, and complex problem solving, supporting learners in meeting real academic and real-world challenges (Dibek et al., 2024).

By enabling learners to analyze information, generate ideas, evaluate alternatives, and tackle complex educational tasks, AI-powered smart assistants can contribute to the development of digital entrepreneurship and future-oriented problem-solving skills, both crucial cognitive skills for university students. They also help cultivate analytical, deductive, and creative thinking abilities, making them a promising tool for preparing learners to address future challenges and the demands of a knowledge-based and innovative-driven digital economy. While AI-powered smart assistants offer significant opportunities to enhance teaching and learning, their use in education presents important challenges. Recent studies have warned that over-reliance on AI-generated responses may impair students’ critical thinking, independent reasoning, and higher-order cognitive skills if these technologies are used without appropriate pedagogical guidance (Zhai et al., 2024). Similarly, researchers at MIT have emphasized the need to integrate generative AI into learning environments in ways that foster critical thinking, encourage the validation of AI outputs, and support active learner participation rather than passive reliance on automated responses (MIT Open Learning, 2024). Therefore, current research advocates for employing AI-powered smart assistants as cognitive partners within well-designed learning environments that promote higher-order cognitive development, rather than replacing human reasoning (Daniel et al., 2025).

Among these modern applications of artificial intelligence, the role of AI-powered smart assistants stands out. These assistants, designed to support university students, aim to develop their digital entrepreneurial skills and enhance their ability to solve future problems creatively and innovatively (Brown et al., 2024). AI-powered smart assistants are defined as AI-based systems designed to support students in developing digital skills such as creating presentations, educational content, videos, and developing online stores, websites, and applications, among other digital skills. They provide intelligent guidance and personalized support to each student in real time (Ahmed, 2024).

With increasing technological advancements, entrepreneurial and problem-solving skills have become key requirements in the modern job market. Smart learning tools can enhance these skills by providing an interactive learning environment based on quantitative data analysis and realistic learning scenarios. Through the use of these tools, university students can acquire critical and creative thinking skills, which can contribute to their preparedness to face future challenges effectively (Anderson & Patel, 2024). Focusing on digital entrepreneurship is an important area for school students in general and university students in particular. It involves establishing small, emerging, and innovative digital projects for university entrepreneurs through careful planning that links university education outcomes with labor market needs, providing job opportunities and reducing unemployment (Mohammed & Hassan, 2024).

Digital entrepreneurship has become a core competency in higher education, requiring students to integrate technological knowledge, creativity, innovation, and problem-solving skills to develop sustainable digital solutions. In this context, AI-powered smart assistants provide learners with opportunities to generate innovative ideas, design digital products, evaluate entrepreneurial alternatives, and refine business concepts through continuous feedback and intelligent guidance. Rather than replacing human creativity, these technologies foster entrepreneurial thinking by supporting opportunity identification, decision-making, collaboration, and iterative product development. Recent evidence suggests that integrating generative AI into entrepreneurship education can enhance students’ entrepreneurial competencies and improve their ability to transform creative ideas into practical digital innovations. Thus, AI-powered smart assistants represent valuable educational tools for promoting digital entrepreneurship and preparing students for rapidly evolving digital economies (Crompton & Burke, 2023; Ouyang et al., 2023; Zawacki-Richter et al., 2024).

Abdul Aziz (2024) pointed out that despite the widespread use and application of artificial intelligence (AI) globally, particularly in business and industry, its use in education remains haphazard. This is due to the lack of a clear educational and applied vision for educational models that support learning through AI applications. Therefore, the study by Gupta and Jaiswal (2025) indicated that AI applications and their various tools are often used merely as content support rather than as part of a learning design based on digital educational tasks. This weakens its effectiveness in building real-world skills for university students to address real-world challenges. Despite this progress in the field of AI, there is a deficiency in integrating it into purposeful educational designs that focus on developing skills and entrepreneurial digital skills. The study by Omeh et al. (2025) concluded that most university students’ use of artificial intelligence applications focuses on general tasks such as paraphrasing, translation, information retrieval, and test design, without actively employing entrepreneurial thinking or future-oriented problem-solving. The findings of the study by Zhu et al. (2024) confirm that 81% of students use AI without a clear understanding of how to leverage it to develop their digital entrepreneurial skills. Furthermore, the study concluded that the consumer use of AI applications does not produce a long-term cognitive impact.

Despite the tremendous growth in training, education, and curriculum development within entrepreneurship institutions, there remains a weak focus on the importance of policies and training programs that support digital entrepreneurship. If a university institution wants to cultivate a culture of digital entrepreneurship, it must begin by adopting appropriate programs and policies within its educational institutions to support it (Hussein, 2023). Furthermore, some studies have focused on university curricula, indicating that most of these curricula still lack the integration of artificial intelligence in developing digital entrepreneurial skills, which include producing a digital product or addressing a real-world digital issue using innovative digital solutions. This was confirmed by the study by Abdulrahman and Talal (2024). The UNESCO (UNESCO, 2024) also pointed out that universities focus on teaching AI applications to teachers while neglecting training in designing student-centered content to develop their skills and abilities in analyzing future problems.

Most recent research points to the role of artificial intelligence in developing self-learning skills and improving student experience, although the focus is often on traditional academic aspects such as improving performance in different subjects and increasing achievement and success rates. However, the impact of these tools on developing digital entrepreneurial skills and solving future problems has not been adequately addressed, especially with regard to artificial intelligence applications that help students deal with the challenges of digital acceleration and digital entrepreneurship (Williams et al., 2024).

Future-oriented problem-solving skills are among the key skills university students must acquire to keep pace with labor market developments and upcoming technological challenges. Artificial intelligence can be used as an analytical tool to help train university students in critical and creative thinking by analyzing problems and providing predictive data-driven solutions (Williams et al., 2024).

Recent advances in generative artificial intelligence (AI) have significantly expanded the potential of AI-powered intelligent assistants to support future problem-solving in higher education. By engaging learners in interactive dialogue, providing adaptive feedback, and facilitating scenario-based learning, these intelligent systems encourage students to anticipate future challenges, evaluate multiple alternatives, develop strategic plans, and make informed decisions in complex and uncertain contexts. Rather than providing ready-made solutions, AI-powered intelligent assistants can facilitate reflective inquiry, predictive reasoning, and evidence-based decision-making, thereby fostering the higher-order cognitive processes necessary for solving future problems. Recent pilot studies and systematic reviews have demonstrated that the purposeful integration of generative AI in higher education enhances learners’ ability to solve complex problems, develop insight, and adapt to rapidly changing technological and social environments (Zhai et al., 2024; Daniel et al., 2025; Y. Zhao et al., 2025). These findings highlight the growing educational value of AI-powered smart assistants in preparing university students with the competencies needed to meet future challenges and succeed in knowledge-based economies.

Ayoub (2015) indicated that the low level of student output in terms of creative and entrepreneurial ideas, or the presentation of projects to solve daily and future problems, is due to the weak attention educational institutions pay to the practical design aspect and problem-solving in real-life situations, compared to their heavy focus on academic achievement. Al-Rumaidi (2018) also concluded that the university has a significant lack of interest in instilling a culture of digital creativity among its students, and there are no strategies to motivate creative students to encourage them to take initiatives. Furthermore, the university’s vision and mission do not encourage the development of creative skills in digital products and projects among its students.

Despite the importance of anticipating and addressing future problems, a key skill of the digital age, the impact of using smart digital assistants on enhancing critical and proactive thinking among university students has not been sufficiently researched. Artificial intelligence can provide analytical applications that help students predict future problems; however, comprehensive studies evaluating the effectiveness of these applications in academic settings have not been conducted (Zhang & Miller, 2025).

Recent evidence suggests that AI-powered intelligent assistants have evolved from mere information retrieval tools into cognitive partners that effectively support the development of higher-order cognitive skills in higher education. Through interactive dialogue, adaptive feedback, and personalized learning support, these technologies encourage learners to engage in critical thinking, analytical reasoning, creative idea generation, cognitive reflection, and complex problem-solving. Recent meta-analyses and systematic reviews consistently demonstrate that generative AI can positively impact higher-order thinking skills when integrated into well-designed learning environments that foster active cognitive engagement rather than passive reliance on AI-generated responses (Dibek et al., 2024; Daniel et al., 2025; Patrick et al., 2025). Consequently, AI-powered intelligent assistants are increasingly viewed as educational technologies capable of fostering the advanced cognitive competencies necessary for innovation, digital entrepreneurship, and future-proofing higher education.

Despite the growing body of research on artificial intelligence in higher education, current studies have primarily focused on overall academic outcomes, technology acceptance, learning performance, or individual cognitive skills (Ouyang et al., 2023; Zawacki-Richter et al., 2024). Relatively little attention has been paid to investigating how AI-enabled intelligent assistants simultaneously enhance higher-order cognitive abilities by fostering digital entrepreneurship and future-oriented problem-solving within a unified educational framework (Daniel et al., 2025). Furthermore, few pilot studies have explored these relationships using structured educational interventions in real-world higher education settings, highlighting the need for more comprehensive evidence regarding the cognitive and educational impact of AI-enabled intelligent assistants (Zawacki-Richter et al., 2024; Daniel et al., 2025). In an effort to bridge this gap, this study examines the effectiveness of AI-powered intelligent assistants in enhancing higher-order cognitive abilities by fostering digital entrepreneurship and future-oriented problem-solving skills among graduate students. By integrating these concepts into a unified conceptual and empirical framework, this study expands existing knowledge on AI-assisted learning and provides practical evidence for designing and implementing intelligent learning environments that promote advanced cognitive development (Ouyang et al., 2023; Daniel et al., 2025).

This study is based on the premise that AI-powered smart assistants can act as cognitive partners, actively supporting learners’ higher-order thinking processes rather than simply facilitating access to information. By assisting learners in organizing knowledge, analyzing complex situations, generating innovative ideas, evaluating alternative solutions, and structuring their learning processes, these intelligent systems can enhance core cognitive functions associated with applied intelligence. In this context, digital entrepreneurship and future-oriented problem-solving are viewed not merely as professional competencies, but as manifestations of complex cognitive skills encompassing reasoning, planning, forecasting, decision-making, creativity, and adaptive thinking.

Accordingly, this study examines the extent to which AI-powered smart assistants contribute to the development of these cognitive abilities among graduate students, thereby contributing to the emerging literature on AI-assisted cognitive development and providing empirical evidence of the role of intelligent educational technologies in promoting higher-order cognitive abilities in higher education.

## 2. Research Problem

With the rapid expansion of the digital revolution and the shift towards a knowledge-based economy, university students are increasingly expected to possess advanced cognitive competencies that enable them to adapt to complex and constantly evolving work environments. Among these competencies, Higher-order cognitive abilities, including analytical thinking, creativity, decision-making, predictive thinking, and future-oriented problem-solving, are considered essential for digital entrepreneurship, innovation, and high performance in dynamic environments. These abilities are not limited to technical skills but represent complex cognitive capabilities that enable learners to anticipate emerging challenges, evaluate alternatives, and respond effectively to future demands. Despite their importance, recent studies have indicated a significant gap among university students in acquiring these competencies, particularly with regard to digital entrepreneurship and the use of predictive thinking to address future problems (Omeh et al., 2025; Zhu et al., 2024). This gap underscores the need to adopt innovative educational approaches capable of developing higher cognitive skills through purposeful learning experiences supported by modern technologies.

The study by Sajja et al. (2024) indicated that university students often lack digital innovation skills, future-oriented problem-solving abilities, and the capacity to make sound, logical, and well-informed decisions. The study also pointed out that traditional learning environments—despite the use of some technological tools—remain unable to meet the future-oriented skills requirements of learners. This weakens their readiness to interact effectively and efficiently with the demands of a labor market based on artificial intelligence applications and smart digital transformation. A study by Mohammed and Hassan (2024) concluded that university students rarely participate in digital entrepreneurial tasks and projects to develop their entrepreneurial thinking. The study also found that only 21% of students possess the ability to solve future-oriented problems.

Kagan (2023) emphasizes that most digital products in universities merely convert traditional content into a digital format. Consequently, learners believe they possess digital creativity skills, but in reality, they know very little about it. In this regard, Al-Khadisi et al. (2025) point out that Arab universities do not pay much attention to digital creativity, focusing instead on proficiency in exams or a culture of submission rather than innovation.

A survey of (10,000) university students from (28) countries showed that (86%) of university students use artificial intelligence in their studies, but the vast majority of them use it only for: research, summarizing, and accessing information, and not for developing entrepreneurial thinking skills or solving future problems. (62%) of the participants indicated that they use artificial intelligence tools without having a clear understanding of how to use them to solve future problems, and (82%) confirmed their need for targeted training in this field (Digital Education Council, 2024).

A report by the National Association of Colleges and Employers (NACE) confirms that 90% of employers seek recent graduates with future-oriented problem-solving skills (National Association of Colleges and Employers, 2024). However, students often lack these skills, leaving them ill-prepared for the job market. This reveals a significant and clear gap between university education outcomes and labor market demands (NACE). While AI-powered smart assistants offer an opportunity to reshape academic support methods, many university learning environments have yet to invest in designing these tools pedagogically to foster digital entrepreneurial skills and future-oriented problem-solving abilities among university students. In fact, some current uses, if not scientifically sound, may lead to increased passive reliance on AI and diminish the active roles of learners (Xing et al., 2025).

In an exploratory study conducted on a sample of (15) graduate students regarding the use of artificial intelligence (AI) applications in education, all students (100%) agreed on the importance of AI and indicated that they own and use AI applications daily for routine educational tasks. However, all (100%) of the students indicated that they lack sufficient knowledge about how to use these applications to solve future problems and that they desire structured training to understand how to employ these applications in entrepreneurial educational activities and future problem-solving.

Based on a review of the literature, previous empirical studies, and exploratory research findings, the current research problem is identified as a deficiency in higher-order cognitive skills among university students, skills that form the basis for digital entrepreneurship and future-oriented problem-solving. Despite the increasing applications of artificial intelligence (AI) in higher education, it is rarely integrated into pedagogically designed learning environments that foster logical, creative, predictive, and decision-making thinking, among other higher-order cognitive processes. Therefore, there is a need to investigate how AI-powered smart assistants can be used as cognitive teaching tools to enhance these complex cognitive skills. Accordingly, this study examines the effectiveness of AI-powered smart assistants in developing the cognitive competencies that underpin digital entrepreneurship and future-oriented problem-solving among graduate students.

## 3. Research Questions

This research attempts to answer the following questions:

1- What is the instructional design model for AI-powered smart assistants in an e-learning environment, aimed at developing digital entrepreneurship skills and future-oriented cognitive problem-solving skills among university students?
2- What is the impact of AI-powered smart assistants developing digital entrepreneurship skills among university students?
3- What is the impact of AI-powered smart assistants on developing future-oriented problem-solving skills as a complex cognitive skill among university students?

## 4. Research Hypotheses

The current research attempted to verify the following hypotheses:

**H1.**  *There is no statistically significant effect at the 0.05 level of AI-powered smart assistants on the development of knowledge-based digital entrepreneurship skills among graduate students, as measured by the Digital Entrepreneurship Product Evaluation Scale.*

**H2.**  *There is no statistically significant effect at the 0.05 level of AI-powered smart assistants on the development of future-oriented problem-solving skills, as a complex cognitive skill, among graduate students, as measured by the Future-Oriented Problem-Solving Scale.*

## 5. Research Objectives

The current study aimed to investigate the effectiveness of AI-powered smart assistants in developing digital entrepreneurship and future problem-solving skills, which are complex cognitive skills among graduate students at the College of Education, through the design of an electronic learning environment supported by AI-powered smart assistants that rely on AI technologies.

## 6. Importance of Research

This research gains scientific and practical importance in light of the rapid and significant digital transformations and the growing need to prepare a generation capable of adapting to future challenges. Therefore, the importance of this research lies in the following:

1- This research provides a practical framework for higher education institutions and curriculum developers to integrate AI-powered smart assistants into learning environments, thereby supporting the development of digital entrepreneurship and higher-order cognitive skills among university students.
2- The research contributes to enriching scientific knowledge related to the educational applications of AI by demonstrating how AI-powered smart assistants can contribute to developing the cognitive competencies necessary for learning, innovation, and decision-making in the digital age.
3- The research findings may help academic leaders and curriculum planners redesign university programs to ensure the integration of AI-based learning tools for developing entrepreneurial thinking, cognitive flexibility, and future-oriented problem-solving skills.
4- The use of AI-powered smart assistants contributes to enhancing critical thinking, analytical reasoning, creativity, and the ability to solve complex problems—essential competencies for success in the current and future job markets.
5- By encouraging students to develop innovative digital solutions and entrepreneurial projects, the research supports the Sustainable Development Goals and contributes to preparing graduates capable of addressing future societal and technological challenges.
6- The findings of this study offer a practical framework that universities can adopt to systematically integrate AI-powered intelligent assistants into higher education. This framework focuses on developing faculty training programs, redesigning curricula to include AI-assisted learning activities, enhancing higher-order cognitive skills through realistic learning tasks, and establishing institutional guidelines for the responsible and effective use of AI in teaching and learning. By adopting these practices, universities can better prepare students for the evolving demands of the digital economy, while fostering innovation and future-proofing their skills.
7- This study contributes significantly to Sustainable Development Goal 9 (Industry, Innovation, and Infrastructure) by promoting the effective educational integration of AI-powered smart assistants to foster innovation, digital entrepreneurship, and technology-enabled learning in higher education. While the study may indirectly support Sustainable Development Goal 4 (Quality Education) by improving teaching practices and learning outcomes, its primary focus is not on inclusive or equitable education.

## 7. Research Literature

### 7.1. Artificial Intelligence and AI-Powered Smart Assistants

The integration of artificial intelligence (AI) and AI-powered smart assistants represents a qualitative leap in the educational process, shifting from a simple, interactive approach to a dynamic, data-driven, and need-based learning environment. AI-powered assistants can improve students’ academic achievement compared to traditional methods. From an educational perspective, this integration marks a shift from directed instruction to personalized learning. AI can analyze students’ learning styles and apply content according to their abilities and preferences, thus reinforcing the concept of smart learning, a rising trend in modern digital education, as confirmed by the study by Sanna et al. (2023).

Artificial intelligence (AI) is theoretically based on several educational theories that link theory and practice, such as activity theory. This theory focuses on how learners acquire and complete educational skills, emphasizing their interaction and thinking during the learning process. It also highlights the importance of providing opportunities for learning practice and repetition to help learners develop their knowledge and skills. Furthermore, this theory emphasizes the importance of engaging learners in digital activities and skills that require them to participate in interactive and immersive learning experiences, such as designing digital skills, websites, applications, online stores, or interactive digital content (Mahmoud, 2024).

Activity theory offers a valuable theoretical framework for understanding learning as a purposeful social process in which learners interact with tools, tasks, and their environment to construct knowledge. Rather than viewing learning as a solitary cognitive activity, the theory emphasizes that purposeful learning arises from dynamic interactions between the learner, technological tools, learning objectives, and the educational context. Accordingly, AI-powered intelligent assistants can be understood as intermediaries that facilitate cognitive sharing, collaboration, and knowledge construction during learning activities.

In this study, activity theory was used to design an educational intervention, where AI-powered intelligent assistants were positioned as intermediaries to support student interaction with learning tasks and promote active participation throughout the learning process. This theoretical perspective also guided the interpretation of the results, demonstrating how structured interactions between learners, AI-powered intelligent assistants, and real-world learning activities contributed to the development of higher-order cognitive abilities, digital entrepreneurship, and future-proof problem-solving skills.

One of the most prominent types of AI is the “intelligent assistant,” a type of AI-powered adaptive support that provides users with specific and useful information when needed to perform a particular task or solve a problem. This is achieved by searching for information related to the topic or problem. That is, AI-powered smart assistants are considered as guidance provided to the learner according to his characteristics and needs in a personal learning environment; with the aim of building a system of aids and guidance that are directed to the learner and his personal environment; and then AI-powered smart assistants become a driver and motivator for building personal learning environments; since everything that is provided to the learner is appropriate to his characteristics, experiences and personal needs (Khalil & Hidayah, 2018).

There is a difference between intelligent assistants and intelligent agents in that an intelligent assistant is primarily designed for interaction, adaptive support, and user input based on understanding their needs, abilities, and guidance. An example of an intelligent agent is a chatbot (ChatGPT 5.4). An intelligent agent, on the other hand, does not require user intervention. It makes decisions based on data and information, and is autonomous, as seen in drones and self-driving cars. In other words, intelligent assistants focus on user interaction and providing immediate assistance in performing tasks, while an intelligent agent operates autonomously and independently, requiring minimal human intervention. It makes decisions autonomously without human intervention.

Lo’s (2023) study concluded that AI-powered smart assistants can be used as tools to support both teachers and students. They can function as virtual teachers, supporting student learning by answering questions, summarizing information, facilitating collaboration and online sharing, assisting in content creation, and providing immediate feedback.

In this regard, Halaweh’s (2023) study concluded that AI-powered smart assistants can be used to develop creative thinking skills. This is achieved by teachers designing digital content on a specific topic, which students then evaluate and verify for accuracy. AI-powered smart assistants can also be used to improve student writing and generate new ideas and information. Sok and Heng’s (2024) study concluded that AI-powered smart assistants offer an opportunity to provide educational support, assess learning, and foster creativity and innovation. Abu Hatab (2024) study focused specifically on smart voice assistants. The study concluded that intelligent voice assistants contribute to enhancing quick and easy communication between learners and technological devices. They can perform skills such as designing emails, providing instant information, answering inquiries, and managing appointments and various other tasks.

Al-Dhabahi (2025) focused on the role of universities in using intelligent assistants in the learning process. He pointed out that universities in the United Kingdom are transforming the traditional student experience into a smart learning experience through the use of chatbots and intelligent academic assistants. This is achieved through student interaction with assistants 24/7, providing students with academic guidance, career counseling, and continuous educational support using natural language processing and machine learning. Intelligent assistants can then provide relevant learning resources and proactive intervention, resulting in students feeling more supported and connected, ultimately leading to more efficient learning and academic success.

It is clear from the above that AI-powered smart assistants are a pivotal technology in developing university education and enhancing its outcomes. They enable university students to organize their thoughts more clearly and generate scientific and creative content that supports critical thinking and innovation skills. They can also contribute to strengthening student-teacher interaction by discussing and analyzing AI-generated information in depth. Furthermore, the importance of these assistants is evident in their ability to empower students to design innovative presentations, generate visuals and diagrams that support projects and research, and even design integrated e-learning modules. Thus, AI-powered smart assistants are a modern cornerstone in preparing university students for the demands of the job market and future technology- and knowledge-based professions. This supports current research trends in designing AI-powered smart assistants to support entrepreneurial education in universities.

In general, previous studies consistently indicate that AI-powered intelligent assistants have significant potential to enhance higher-order cognitive abilities by fostering critical thinking, problem-solving, creativity, and self-directed learning. However, these studies have largely focused on examining these cognitive outcomes in isolation or within specific educational contexts, providing limited evidence on how AI-enabled learning environments can simultaneously enhance multiple higher-order cognitive abilities through structured educational interventions. This gap highlights the need for further empirical research investigating the broader cognitive impact of AI-powered intelligent assistants in real-world higher education settings.

### 7.2. AI-Powered Smart Assistants and Entrepreneurial Digital Skills

Digital skills refer to a range of activities performed using modern digital applications. These skills include, but are not limited to designing educational videos, creating educational applications and smart platforms, producing interactive digital content, and designing educational digital stories. Students’ engagement with these skills can enrich their learning process by fostering and developing active educational participation, thereby improving learning outcomes. Furthermore, using these modern technologies in designing educational skills can help cultivate 21st-century skills such as critical thinking, problem-solving, and collaborative work, preparing students to face future challenges effectively and flexibly. Digital skills can be implemented in both online and in-person learning environments, enhancing student participation and interaction with educational content. Therefore, developing these pioneering digital skills helps students integrate into a technology-driven society, enabling them to interact with and navigate modern advancements with efficiency and advanced digital awareness—in short, preparing them effectively for the job market.

Digital learning skills can be categorized into several types based on their nature and purpose. These include individual digital skills aimed at enhancing understanding and content delivery, such as designing educational videos, creating learning platforms, or producing interactive digital content designed to clarify concepts. There are also digital learning tasks that rely on collaboration and partnership among students, such as collaborative group work to design digital documents or models using Google tools or implementing e-projects through collaborative platforms like Padlet, these foster teamwork and communication skills. Additionally, there are creative digital tasks that focus on developing critical and creative thinking skills, such as producing digital storytelling, analyzing student data, and preparing innovative electronic reports. All these types of digital skills contribute to enhancing learner engagement and developing higher-order thinking skills such as analysis, evaluation, and creativity, thus supporting learning efficiency and preparing students for the job market.

Skills-based learning is considered one of the most important strategic shifts in the field of education. This is because this type of learning relies on skills such as educational and research tools. It also includes individual and group activities and projects that give the learner a greater role in their learning process and allow the teacher to observe student activities during learning. Digital skills-based learning helps build a sense of learning community (Al-Halafawi, 2018).

On the other hand, Mustafa study (Mustafa, 2020) focused on exploratory digital skills and indicated that they rely on experiential learning theory. This is one of the main theories that can be relied upon in performing exploration digital skills. Experiential learning indicates that the methods individuals use to perform skills consist of a set of procedures that are characteristic of the learner in receiving, perceiving, and processing information based on their personal experiences. This can be facilitated through a training environment using smart digital aids, giving the learner the opportunity to practice their personal experience in exploring and completing the task.

Naj (2021) points to an important aspect: when students engage in digital tasks such as designing an e-book, producing an educational video, or designing a user interface, they participate in developing their creative skills. Designing technology-based skills is crucial; teachers have noted that passively sitting in front of a screen reading text or watching a video yields minimal knowledge acquisition. Many students simply click and scroll quickly for a quick read or glance. However, by designing entrepreneurial digital skills, students explore their potential and prepare for entrepreneurial careers in the job market.

Entrepreneurial education in higher education institutions is a modern trend in the field of education. It contributes to developing students’ skills in creating small, innovative startup projects. This type of education aims to enhance planning skills and align university education outcomes with the demands of the modern market, thus contributing to the creation of new job opportunities and reducing unemployment rates after graduation (Mohammed & Hassan, 2024).

Therefore, many countries have adopted creative education in their educational systems to cultivate creativity in their future generations, recognizing it as the primary driver of economic and social development. In the United States, a week each year is dedicated to creative work, in addition to advanced educational programs offered by universities in the field of digital creativity. In Japan, universities have been granted autonomy in creative development, bridging the gap between educational outcomes and labor market needs (Al-Hana’i & Shehat, 2022).

Entrepreneurial digital skills require students to research, experiment, and discover using advanced technological platforms and tools. In this regard, artificial intelligence enables learners to design new models based on big data analysis and the generation of patterns from it (Smith & Lee, 2025). These capabilities allow learners to explore new educational content in innovative ways, as artificial intelligence can analyze scientific materials and provide explanations and summaries tailored to the individual learner’s needs. This enhances interaction with educational content and increases their understanding of complex subjects (Zhu et al., 2024). In this regard, Al-Bahiri (2023) study concluded that the use of artificial intelligence applications contributed to the design of creative digital advertisements and achieved significant breakthroughs and updates in the field of digital advertising design. Similarly, Morris and Tan’s (2024) study concluded that AI-based digital learning environments can help provide opportunities for entrepreneurial learning, enabling learners to design digital solutions applicable in real-world situations. This represents a qualitative shift in the concept of learning from rote memorization to digital creativity and innovation, and the current research aligns with this study.

Intelligent assistants can provide interactive scenarios using virtual and augmented reality technologies, facilitating unconventional learning experiences. These technologies offer learners the opportunity to conduct virtual laboratory experiments, allowing them to explore physical and chemical phenomena in dynamic and safe ways (Johnson & Carter, 2024). Through these simulations, students can test their hypotheses and analyze the impact of their decisions within diverse, realistic scenarios, enhancing their ability to address problems scientifically and practically. Intelligent assistants can also offer solutions based on the analysis of past data, systematically and effectively supporting the development of students’ analytical and predictive thinking skills (Garcia & Kim, 2025).

Intelligent assistants are AI-powered applications that provide immediate support and personalized guidance to learners, potentially helping them develop entrepreneurial skills that enable them to handle digital skills efficiently and effectively (Mohammed & Hassan, 2024). AI-powered smart assistants are distinguished by their ability to perform digital skills such as analyzing big data related to learner performance, allowing for the personalization of the learning experience according to each learner’s needs. A study by Ahmed (2024) indicates that the use of AI-powered smart assistants has significantly contributed to the design of these tools and to improving learners’ academic performance, as they provide intelligent results and guidance based on the analysis of individual learning styles. These digital entrepreneurial skills represent applied manifestations of higher cognitive functions because they require thinking, planning, creativity, decision-making, and adaptive thinking.

From the above, it is clear that university students can use AI-powered smart assistants to develop pioneering digital projects, starting with analyzing available opportunities, understanding labor market needs, and utilizing smart technologies. These technologies not only improve students’ performance at university but also prepare them to face the challenges of a labor market that demands skills in technology, critical thinking, innovation, and entrepreneurship.

Most previous research indicates that AI technologies can support digital entrepreneurship by fostering innovation, opportunity discovery, and entrepreneurial decision-making. However, most published studies have focused on technology adoption or entrepreneurial intent, while few have examined the effectiveness of AI-powered intelligent assistants in developing practical digital entrepreneurial competencies through educational interventions. Therefore, further empirical research is needed to understand how AI can be systematically integrated into higher education to cultivate entrepreneurial capabilities in digitally transformed societies.

### 7.3. Artificial Intelligence and Solving Future Problems

A problem represents a gap between the current state and the desired state. This gap encompasses difficulties and obstacles that an individual faces when transitioning between the two states. These difficulties either prevent or hinder the achievement of a desired level of performance (Al-Feel & Abdel-Hadi, 2014). An individual can solve some problems with minimal effort, relying on prior experience. However, another type of problem requires complex solutions, as it is a problem the human mind has never encountered before, necessitating the development of thinking skills to address it (Wu & Molnár, 2022).

Problem-solving skills are defined as a set of mental processes that an individual employs to overcome any obstacle or problem they encounter through a series of organized steps aimed at resolving that problem or obstacle (Hamed, 2023). Future problems are unclear or undefined difficulties or obstacles expected to occur in the future. They represent the most challenging problems because they are not well-defined or clear to individuals. This requires them to study and analyze the current reality and consider its future implications. Therefore, learners should employ sound methodologies to predict the future, drawing upon their higher-order thinking skills, in order to make informed decisions (Al-Kilani & Al-Zoubi, 2019).

The world today is characterized by a high degree of complexity and rapid change, necessitating a strong capacity for future-oriented thinking and the ability to anticipate potential problems before they occur. It also demands the ability to make informed decisions based on analytical, critical, and creative thinking, thereby enhancing their capacity to adapt to the demands of the future job market, which is characterized by uncertainty and rapid, continuous fluctuations (Du et al., 2025). Critical thinking, innovation, and self-learning are core skills within what are known as future skills. They are closely linked to the ability to solve complex problems, and their importance is particularly evident at the university level, as they serve as a bridge to the job market and research and development fields (Alshammari & Khan, 2024).

The current research aligns with the study by Mohammed and Hassan (2024), which found that future-oriented problem-solving skills help university students understand the future, deal with it more effectively, and develop positive attitudes toward it that contribute to real change. These skills also foster creative and innovative thinking. This study indicated that future-oriented problem-solving skills include visualization, prediction, anticipation, planning, intuition, and imagination.

The study by Baraida and Al-Harbi (2025) indicated that future problem-solving skills consist of: prediction, imagination, visualization, planning, and expressing opinions. Shadeed and Al-Nadhir (2022) focused on another aspect of future problem-solving skills: “information problem-solving.” They indicated that it comprises the skills of identifying skills, information search strategies, locating and accessing information, utilizing information, and synthesis and evaluation.

The study by Al-Ghamdi (2024) linked future problem-solving skills to future thinking skills. It indicated that future problem-solving skills are part of future thinking skills, which include prediction, imagination, anticipation, and future problem-solving. It noted that these skills are acquired by students through learning and enable them to choose desirable alternatives and gain insight into the future and its events.

Future problem-solving education helps university students build strategic capabilities that enable them to deal with problems that have not yet occurred by developing skills in forecasting, strategic planning, and adapting to expected changes. This type of education also enhances university students’ ability to think from a comprehensive perspective, and this may contribute to preparing and helping them to face future challenges related to modern technologies, sustainability, socio-economic changes, and other areas (Folomieieva et al., 2024).

Hafez (2023) noted that ChatGPT (Chat Generative Pre-trainer Transformer) can be a valuable tool for teachers in enhancing future problem-solving skills by leveraging the power of intelligent assistants. ChatGPT provides students with a range of problem-solving activities that help them understand geographical concepts and apply them to real-world issues and challenges. For example, ChatGPT can be used to help students develop data analysis and interpretation skills by providing access to real-world data and tools for data analysis and interpretation. This, in turn, can help students understand the relationship between different geographical factors and their impact on different regions, enabling them to make informed decisions about real-world issues.

Al-Kalbani (2024) focuses on the application of ChatGPT, indicating that teachers can present students with a problem relevant to their students’ lives and the subject matter, prompting them to seek solutions. ChatGPT can also be used to generate problems related to the curriculum, allowing students to research and discuss solutions. Teachers can create small groups where students use ChatGPT to exchange ideas on potential solutions to the problem. With ChatGPT, the ideas and suggestions generated by both the students and the platform can be creative.

Many of the world’s leading universities are undergoing significant transformations in teaching and learning methods by integrating artificial intelligence (AI) to develop students’ future problem-solving skills. Arizona State University, for example, has implemented an AI-powered learning platform called ChatGPT 5.4 Enterprise in collaboration with OpenAI. This platform analyzes student interactions and delivers customized content to help them understand complex concepts through analytical and interactive questions (Lin et al., 2024). At the University of Toronto in Canada, an AI Tutor learning system is used to simulate real-world scenarios, presenting students with open-ended challenges that require critical and collaborative thinking, and enhancing their ability to make data-driven decisions through continuous interaction with AI (Sako, 2024).

It is clear from the above that AI-powered smart assistants are among the modern tools that can revolutionize the development of future problem-solving skills among university students. They can contribute to creating interactive training environments that allow learners to analyze changing scenarios, predict future challenges, and plan to address them in innovative ways. Experiences at prestigious universities such as the University of Arizona and the University of Toronto have shown that integrating AI-powered smart assistants into education has helped students simulate real-life situations and make data-driven decisions, thus confirming the role of these assistants in enhancing cognitive and skill-based preparedness for future changes.

Accordingly, future-oriented problem-solving can be conceived as a high-order cognitive competency that integrates prediction, reasoning, planning, creativity, and evidence-based decision-making.

The reviewed studies, taken together, demonstrate the growing importance of future-oriented problem-solving as a core skill for navigating complex and technologically advanced environments. While previous research has acknowledged the potential of artificial intelligence (AI) to support analytical and predictive thinking, its role in enhancing future-oriented problem-solving through structured learning experiences has received insufficient attention. This highlights the need for pilot studies investigating how AI-powered intelligent assistants can develop students’ ability to anticipate challenges, devise creative solutions, and make informed decisions in future-oriented contexts.

### 7.4. AI-Powered Smart Assistants and Higher-Order Cognitive Development

In contemporary intelligence research, cognitive tools are defined as technologies that enhance learners’ cognitive abilities rather than simply automate learning tasks. These tools support the organization and application of knowledge, and facilitate thinking, planning, prediction, and adaptive decision-making. Accordingly, AI-powered smart assistants represent an emerging category of cognitive technologies capable of enhancing applied intelligence in higher education.

AI-powered smart assistants have transcended their traditional role as information retrieval systems, evolving into cognitive tools that support higher-order thinking processes in learners. Recent research indicates that these assistants can provide personalized and adaptive learning support by analyzing learners’ needs, generating interactive responses, and delivering immediate feedback that enhances engagement and learning efficiency (Sajja et al., 2024). In higher education, these tools are particularly important due to their ability to support knowledge organization, analytical reasoning, decision-making, and reflective thinking. Recent systematic reviews have shown that AI tools are widely used in higher education for intelligent teaching, personalized learning, automated feedback, learning analytics, and educational chat assistants. These applications can enhance cognitive and metacognitive skills when integrated into purposeful instructional designs, rather than being used solely as answer-generating tools (C. Zhao, 2024). Similarly, generative AI tools in higher education have been found to hold significant potential for transforming learning processes, although their effectiveness depends on their thoughtful educational integration and ethical use (Mohammed & Hassan, 2024). 

AI-powered teaching systems and learning assistants can support problem-solving and cognitive development by adapting learning tasks to students’ performance levels and providing necessary support during complex activities. A recent systematic review of AI-enabled intelligent teaching systems confirmed their ability to improve learning outcomes through adaptive support, personalized feedback, and learner-centered interaction (Luo et al., 2025). Furthermore, studies on AI-assisted learning indicate that generative AI can foster critical thinking, creativity, and problem-solving when students are guided to evaluate, justify, and improve AI outputs rather than passively accepting them (Zhang & Miller, 2025; Y. Zhao et al., 2025).

From this perspective, AI-powered smart assistants can be understood as cognitive partners that expand learners’ ability to analyze information, generate ideas, compare alternatives, and make informed decisions. This is particularly relevant to digital entrepreneurship and future problem-solving, as both learners integrate knowledge, anticipate challenges, design innovative solutions, and evaluate potential outcomes. Therefore, the current study views AI-powered smart assistants not only as assistive technology tools, but as cognitive tools that can enhance applied intelligence, entrepreneurial thinking, and complex cognitive skills among university students.

Therefore, this study defines AI-powered smart assistants as cognitive technologies that enhance learners’ Higher-order cognitive abilities by supporting reasoning, knowledge organization, predictive thinking, creativity, and evidence-based decision-making. From this perspective, digital entrepreneurship and future-oriented problem-solving are seen as practical applications of these complex cognitive abilities. Accordingly, this study investigates how the instructional design of AI-powered smart assistants contributes to the development of cognitive abilities and applied intelligence in graduate students.

### 7.5. Conceptual Framework of the Study

This study is based on the premise that AI-enabled intelligent assistants function as cognitive learning tools that support higher-order cognitive abilities through structured instructional design. Within this framework, higher-order cognitive abilities constitute the core of the study, while digital entrepreneurship and future-oriented problem-solving are considered measurable manifestations of these abilities. Instructional design provides the pedagogical framework through which AI-enabled intelligent assistants facilitate learning, while cognitive partnership, adaptive learning, and applied intelligence are considered complementary theoretical perspectives that explain the mechanisms by which AI-assisted learning can enhance students’ cognitive performance. Accordingly, the experimental portion of this study specifically focuses on examining the effects of AI-enabled intelligent assistants on digital entrepreneurship and future-oriented problem-solving in higher education.

Accordingly, although many complementary theoretical perspectives have been discussed to explain AI-assisted learning, the empirical focus of the current study is limited to examining the effects of AI-assisted intelligent assistants on two top-order cognitive outcomes: digital entrepreneurship and future-oriented problem-solving.

## 8. Materials and Methods

This section describes the methodological procedures adopted in the study, including research design, participants, AI-supported educational intervention, research instruments, study procedures, and statistical analyses conducted to address the research objectives and test the study hypotheses.

### 8.1. Research Design

The current research employed a quasi-experimental design based on a two-group approach with pre- and post-testing of the research instruments.

### 8.2. Participants and Research Context

To assess the impact of AI-powered smart assistants on developing digital entrepreneurship and future-oriented problem-solving skills among graduate students at a higher education institution, the following procedures were followed:

First: Selection of the research population and sample: The research population consisted of 180 master’s students enrolled at a higher education institution. Purposeful sampling was used to select a random sample of 82 students (45%), who were randomly divided into two groups: an experimental group of 41 students trained using the Canvas -2025 platform with the assistance of AI-powered smart assistants, and a control group of 41 students trained using only the Canvas platform. Participants were selected because they were enrolled on practical courses in educational technology. To ensure the equivalence of the two groups, the research instruments were administered as a pre-test, and the results are shown in Table 1.

Table 1 shows that the calculated (t) values (1.11) and (1.60) in the product evaluation card and the future problem-solving skills scale are all non-significant, at the level of (0.05), which shows that there is no statistically significant difference between the two groups in the pre-application of the product evaluation card and the future problem-solving scale, which shows the equivalence of the two groups.

### 8.3. Learning Activities and Student Interaction

During each learning session, students in the pilot group actively interacted with AI-powered smart assistants to perform a variety of learning tasks. These tasks included generating ideas, analyzing learning problems, developing digital products, refining answers through repeated guidance, and receiving immediate feedback. Students were encouraged to critically evaluate the generated outputs, compare alternative solutions, and adjust their guidance to obtain more accurate and appropriate answers. The instructor supervised these activities, provided guidance when needed, and ensured that the AI tools were used to support, not replace, students’ independent thinking and decision-making abilities.

### 8.4. AI Prompting Procedures

To ensure consistency in learning activities, students were guided to use structured instructions when interacting with AI-powered intelligent assistants. These instructions were designed to stimulate higher-order cognitive processes, encourage creative thinking, and support digital entrepreneurship and future-oriented problem-solving. While students were allowed to refine and expand their instructions according to the learning task, the educational framework emphasized frequent instruction, critical evaluation of AI-generated responses, and continuous improvement of outcomes based on academic requirements.

### 8.5. Intervention Design and Implementation

The intervention was implemented during a structured learning period, in which both the experimental and control groups studied the same educational content, completed identical learning activities, and were assessed using the same evaluation criteria. The only difference between the two groups was the instructional support provided. Students in the experimental group completed all learning activities using AI-powered smart assistants integrated into the Canvas learning environment 2025, while students in the control group completed the same activities through traditional instruction using the Canvas platform without AI assistance. Throughout the intervention period, the teacher followed the same teaching schedule, learning objectives, and assessment procedures for both groups to ensure consistency and minimize potential extraneous variables.

### 8.6. AI-Powered Learning Intervention

Designing a Learning Environment Based on AI-powered smart assistants:

To design a learning environment based on AI-powered smart assistants, some previous studies were reviewed, such as those by Abu Hatab (2024) and Sako (2024). The ADDIE general design model was as follows:

First Stage: Analysis

The following procedures were carried out in this stage:

- Defining the general objectives of the learning environment based on AI-powered smart assistants; the general objective of this environment is to develop entrepreneurial digital skills and future problem-solving abilities among the research sample.
- Learner Characteristics: The participants were graduate students at the College of Education at a higher education institution, in the first semester of the academic year 2024/2025. They came from a similar environment with comparable circumstances, and their computer and internet skills were nearly identical. The first experimental group consisted of 41 students, and the control group also consisted of 41 students.
- Training Material: The training content was defined as five training units.

Phase Two: Design Phase

The design phase includes defining the procedural objectives for the learning environment based on AI-powered smart assistants, developing a comprehensive vision for the content, learning strategy, appropriate activities, and assessment methods, as follows:

A- Procedural Objectives for the Learning Environment Based on AI-powered smart assistants:

First Topic: Designing a Chatbot

Upon completion of this content, the student should be able to:
▪Distinguishes between information-based chatbots and those that rely on artificial intelligence.▪Designing a conversation scenario using a generative artificial intelligence platform▪Collaborate with classmates to develop a group chatbot.▪It explains the tools and platforms used in building chatbots.

Topic Two: Designing an Intelligent Assistant for Designing Electronic Content:

Upon completion of this content, the student should be able to:
▪Discuss what a smart assistant is.▪Designs a model for a smart assistant to create electronic content.▪Employing artificial intelligence tools such as ChatGPT or Canva 2025 AI in designing an interactive digital lesson▪It adheres to the principles of privacy and security when designing a smart assistant that handles student data.

Third topic: Designing a smart learning platform:

After completing this content, the student should be able to:
▪This section discusses the nature of a smart learning platform.▪Differentiates between traditional and smart learning platforms.▪It designs a model for a smart online platform.▪Creates a dashboard that enables the teacher to track student progress.

Topic 4: Designing a Data Analysis Tool:

Upon completion of this content, the student should be able to:
▪Discusses the concept of an intelligent assistant for data analysis.▪It differentiates between traditional data analysis tools and AI-powered smart assistants.▪Designs a model for an intelligent assistant that analyzes educational data.▪It explains the importance of artificial intelligence in supporting data-driven decision-making.

Topic 5: Designing an intelligent virtual assistant to solve future problems:

After completing this lesson, the student should be able to:
▪Discusses the nature of a “smart virtual assistant” to address future challenges.▪Explains the differences between virtual and future assistants.▪Designs a smart assistant that can handle future scenarios.▪It employs artificial intelligence applications such as (ChatGPT API) in a virtual assistant project.

Learning Environment Content:

The content of the smart aid-based learning environment included the following topics:
▪Topic 1: Designing a Chatbot▪Topic Two: Designing an intelligent assistant for designing electronic content.▪Topic 3: Designing a Smart Learning Platform.▪Topic Four: Designing an assistant for data analysis.▪Topic Five: Designing an intelligent virtual assistant to solve future problems.

Learning Strategy and Activities in the AI-powered smart assistants-Based Learning Environment:

In light of the procedural objectives and the content of the learning environment, an AI-powered smart assistant-based learning strategy was implemented according to a self-directed adaptive learning model supported by artificial intelligence technologies. All practical and training activities were conducted using the computers available at the college, with direct monitoring of skill development and results.

Assessment Methods:

Assessment methods varied, including pre-assessment at the beginning of each topic to review prior learning; formative assessment during each module to guide student learning and provide feedback; and summative assessment, which takes place after the completion of all training content designed using AI-powered smart assistants, to assess the development of digital entrepreneurial skills and future problem-solving abilities.

Phase Three: Development Phase

In this phase, the following AI-powered smart assistants were used:

1- Edu Chat Bot for designing a chatbot.
2- EduBot, a smart educational assistant that helps with content creation and design.
3- SmartLearn for designing a smart learning platform.
4- SmartAnalyst for analyzing educational data.
5- FutureSolver for solving future problems.

The AI-powered smart assistants were designed using GPTs Builder, with each assistant containing: a name, description, language, functions, publishing settings, and the ability to publish the assistant as a link or QR code. The names of the AI-powered smart assistants for the previous topics were as follows:
Names of the AssistantsHelper Link▪Smart assistant for designing a chatbothttps://chatgpt.com/g/g-68077f1221588191b247895a3894e042-sn-rwbwt-ldrdsh-ldhky (accessed on 24 February 2026)▪A smart assistant that helps with content production and designhttps://chatgpt.com/g/g-68072bbaa0048191bb2e0195f2634649-ms-d-dhky-ltsmym-mhtw-lktrwny-tlb-drst-l-ly (accessed on 24 February 2026)▪Smart assistant for designing a smart educational platformhttps://chatgpt.com/g/g-680746d5964881919350bdc45184d871-ms-d-dhk-ltsmym-lmnst-lt-lymy-ldhky (accessed on 24 February 2026)▪Smart assistant for analyzing educational datahttps://chatgpt.com/g/g-6807829318f081918b3164ea97f1915d-mhll-lbynt-lt-lymy-ldhky (accessed on 24 February 2026)▪A smart assistant for solving future problemshttps://chatgpt.com/g/g-6806a2054fd881919fbd8d497ad7f65c-mhll-lmstqbl-ldhky (accessed on 24 February 2026)

All the AI-powered smart assistants were specially developed by the researchers using OpenAI GPT Builder and remained available throughout the intervention period.

Phase Four: Implementation Phase

In this phase, the AI-powered smart assistant-based e-learning content was implemented with (41) users. Instructions were also provided on how to access these assistants and the required digital skills.

Phase Five: Evaluation Phase

In this phase, the training content designed for AI-powered smart assistants was presented to a group of specialists in computer science, artificial intelligence, and educational technology. The research tools, namely a digital product evaluation form and a future problem-solving scale, were also applied after reviewing all the training content with the students in the research sample.

### 8.7. Instruments

#### 8.7.1. Product Evaluation Card Preparation: This Card Was Prepared According to the Following Steps

The purpose of the evaluation card:

The evaluation card aimed to assess the innovative digital product designed by graduate students at the College of Education.

Card dimensions: After reviewing research and studies focused on the field of digital entrepreneurial skills, the card’s main sections were identified, totaling (10) sections. These sections covered a range of topics, including: the originality of the idea, the quality of the design and its entrepreneurial value, its relevance to labor market needs, the presentation of the task, the overall appearance of the product and its functionality across all mobile devices and browsers, user experience, and product sustainability.

Presenting the Initial Card to a Panel of Experts: After finalizing the initial version of the card, it was presented to experts specializing in information technology, educational technology, and psychology. Their feedback confirmed the card’s suitability for the research sample, while noting some points that necessitated revising several paragraphs to enhance clarity and accuracy.

Pilot Application of the Card: Following the panel’s feedback, the card was piloted with a sample of 21 graduate students from the College of Education at a higher education institution to assess the accuracy of its linguistic formulation, its suitability for the students, and to calculate its reliability.

Calculating Card Reliability: After presenting the card to a panel of experts and piloting it with 21 students, the card’s reliability was calculated using the Cooper coefficient. It was found to be approximately 0.92, which is considered a suitable reliability ratio for the card. To ensure evaluation consistency, each student’s digital entrepreneurial product was independently evaluated by trained assessors using the same standardized evaluation criteria and performance indicators. Prior to the evaluation process, assessors were briefed on the evaluation criteria and their corresponding performance indicators to ensure a consistent interpretation. The level of agreement among assessors was calculated, demonstrating a high degree of evaluation consistency and supporting the reliability of the product evaluation.

The digital products were independently evaluated by two qualified reviewers using the same standardized evaluation criteria and rules. To minimize potential bias, each reviewer assessed the products independently, and final scores were determined based on agreed-upon evaluation procedures. Inter-reviewer reliability was assessed using the Cooper Coefficient of Agreement, which was 0.92, indicating a high level of agreement between the reviewers.

The final version of the card: After drafting the card, presenting it to a group of arbitrators and experts, and adjusting it statistically, the card became suitable for final application.

#### 8.7.2. Prepare a Scale for Solving Future Problems According to the Following Steps

Defining the Scale’s Objective:

The scale aims to measure the future problem-solving skills of graduate students at the College of Education at a higher education institution. After reviewing several studies, such as those by Ayoub (2015) and Mohammed and Hassan (2024), the scale’s dimensions were identified as follows: visualization, prediction, anticipation, and planning.

Identifying Future Challenges:

The anticipated future technological challenges were identified based on previous studies and research, as well as operational definitions. This list was then presented to a group of specialists in information technology and educational technology. Following its approval, the following challenges were identified: the digital divide, digital acquisition, over-reliance on technology, remote electronic assessment, the quality of digital content, recognition of digital certificates, information security and data privacy, digital burnout, teacher training, and digital bias.

Scale items:

The scale consists of four dimensions: the perception dimension and the number of its items (6), the prediction dimension and the number of its items (6), the expectation dimension and the number of its items (6), and the planning dimension and the number of its items (6), thus the total number of scale items became (24).

The scale was refined through:

Presenting the initial version of the scale to a group of experts: After finalizing the scale items, it was presented to a group of specialists in educational technology, curriculum, and psychology. Their feedback confirmed the scale’s suitability for its intended purpose, with some linguistic revisions suggested.

Pilot application of the scale:

The scale was administered to a pilot sample of (21) graduate students at the College of Education at a higher education institution, to assess the linguistic and scientific suitability of the items. Their responses confirmed the suitability of the scale items from both a linguistic and scientific perspective.

Internal consistency of the scale (statistical validity):

The Pearson correlation coefficient matrix was found between the scale dimensions and the total score according to the following table:

It is clear from the above that the correlation coefficient of the first dimension with the scale as a whole is (0.743), the correlation coefficient of the second dimension with the scale as a whole is (0.663), the correlation coefficient of the third dimension with the scale as a whole is (0.794), and the correlation coefficient of the fourth dimension with the scale as a whole is (0.669), and with the scale as a whole is (0.67). All of these values are statistically significant and acceptable. This indicates that the dimensions of the scale measure the same thing as the scale as a whole measure, which demonstrates the validity of the scale and its dimensions.

Calculating the average time of the scale: The time of the scale was calculated by finding the average time of all students, each according to their speed, and it was approximately (25) minutes.

Calculating the reliability of the scale: After presenting the scale to a group of experts and piloting it with (21) students, the reliability of the scale was calculated using the SPSS version 30 method. It is more accurate than Cronbach’s alpha, and its reliability coefficient was found to be approximately 0.86, which is a suitable reliability coefficient.

Final version of the scale: After formulating and statistically adjusting the scale, it is now ready for final application.

Pre-application of research instruments:

The research instruments, namely: a digital product evaluation form and a future problem-solving scale in the field of educational technology, were administered to the research group during the first semester of the academic year 2024/2025.

Implementation of the research experiment:

After clarifying the objective of the experiment, the research experiment was conducted during the first semester of the academic year 2024 at the College of Education over approximately five weeks. The research group consisted of 41 students who were trained using AI-powered smart assistants.

Post-application of measurement instruments:

Following the completion of the research experiment, the measurement instruments, namely: a digital product evaluation form and a future problem-solving scale, were administered both before and after the research groups, and the results were recorded and analyzed.

## 9. Results

After recording the students’ scores in the post-test in both: the Digital Entrepreneurial Product Evaluation Card and the Future Problem-Solving Scale, the research questions were answered as follows:

### 9.1. Answer to the First Question, Which Stated: What Is the Instructional Design Model for AI-Powered Smart Assistants in an e-Learning Environment, Aimed at Developing Digital Entrepreneurship Skills and Future-Oriented Cognitive Problem-Solving Skills Among University Students?

To answer this question, a review of literature and studies focusing on smart environments in general and AI-powered smart assistants in particular was conducted, including studies by Al-Bahiri (2023), Halaweh (2023), and Lo (2023).

**Figure 1:**
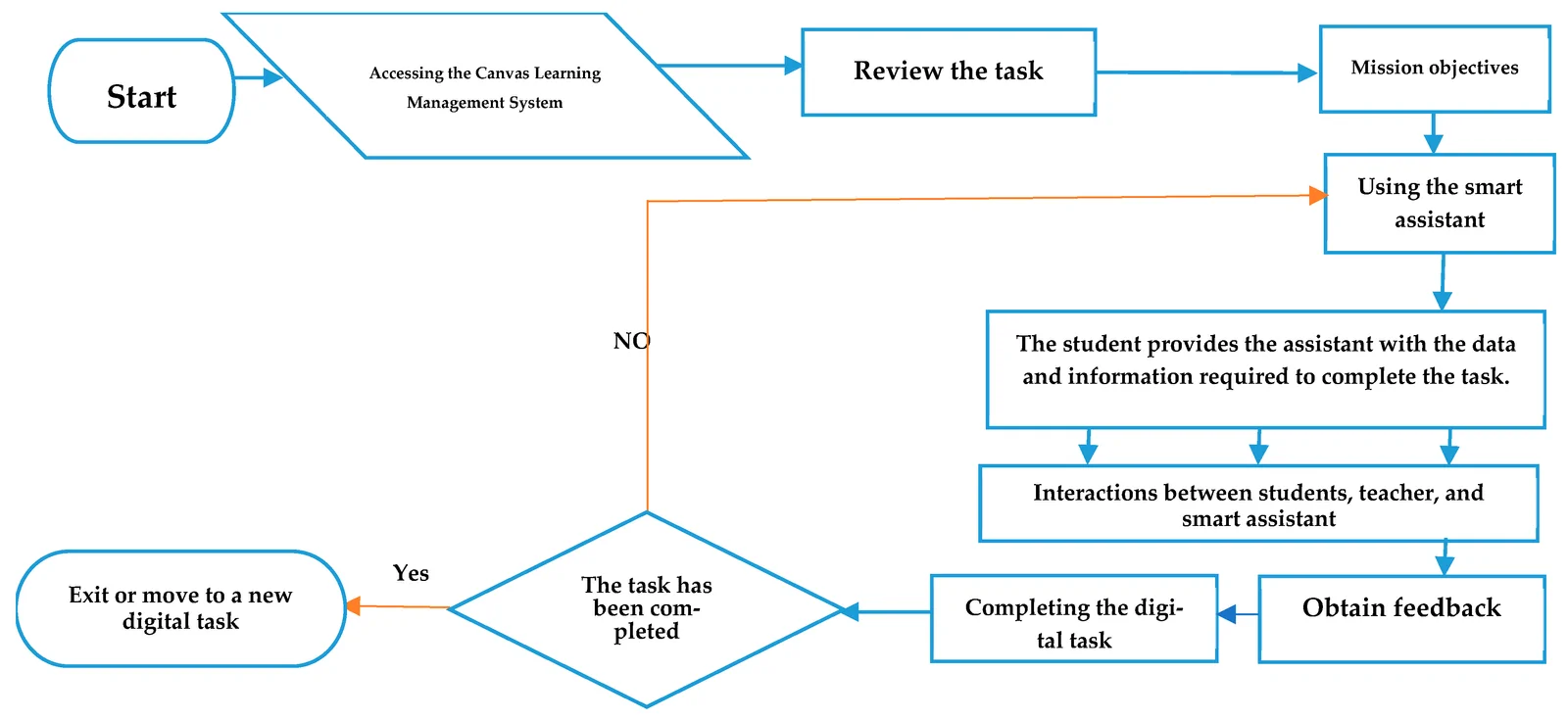
Flowchart in an e-learning environment based on AI-powered smart assistants.

The proposed e-learning environment was validated by a committee of educational technology and information technology specialists and piloted with a sample of graduate students. After necessary improvements, the AI-powered, smart assistant-based e-learning environment was deemed ready for implementation with the main research sample. Figure 1 illustrates the environment’s workflow diagram. Thus, the first research question was answered.

The content was designed according to a general design model that incorporates the use of AI-powered smart assistants. The stages of this model have been previously explained in detail. The content of this e-learning environment was approved after being presented to a group of specialists in educational technology and information technology. It was also piloted with a sample of graduate students. The resulting e-learning environment based on AI-powered smart assistants was then ready for implementation with the main research sample. The flowchart of this environment is shown in Figure 1. This answers the first research question.

### 9.2. Answering the Second Question, Which Stated: What Is the Impact of AI-Powered Smart Assistants Developing Digital Entrepreneurship Skills Among University Students? 

To answer this question, the following hypothesis was formulated: There is no statistically significant effect at the 0.05 level of AI-powered smart assistants on the development of knowledge-based digital entrepreneurship skills among graduate students, as measured by the Digital Entrepreneurship Product Evaluation Scale.

To test the validity of this hypothesis, statistical analysis was conducted using an independent samples *t*-test to compare the scores of the experimental and control groups on the entrepreneurial digital product evaluation card. Table 2 shows the results of applying the *t*-test to the significance of the differences between the mean scores of the experimental and control groups on the entrepreneurial digital product evaluation card.

Table 3 shows that the calculated t-value (9.188) is significant at the (0.05) level, with two sides and (80) degrees of freedom. This indicates a statistically significant difference between the experimental and control groups in the post-test of the digital entrepreneurial product evaluation card.

**Figure 2:**
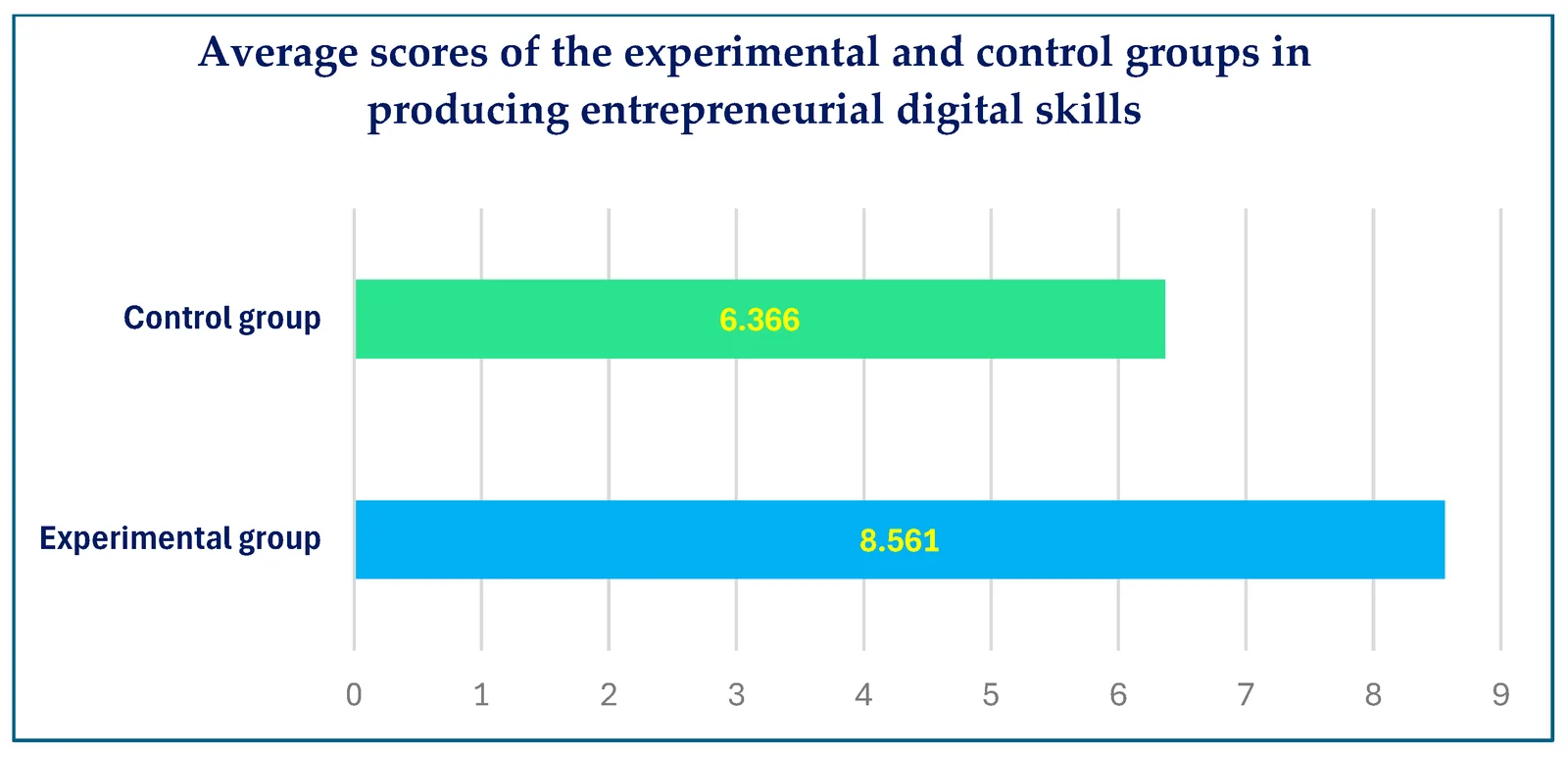
Shows the difference between the experimental and control groups in acquiring digital entrepreneurship skills.

Therefore, the first research hypothesis was rejected due to a statistically significant difference at the (0.05) level between the mean scores of the experimental and control groups on the post-test of the Digital Entrepreneurship Product Evaluation Card for graduate students, in favor of the experimental group that used AI-powered digital tools. Figure 2 illustrates the difference between the experimental and control groups in acquiring digital entrepreneurship skills.

The aforementioned result, attributed to the use of AI-powered smart assistants, may have provided students with immediate feedback as they worked on the digital task. This was achieved by directly assessing their progress, enabling them to identify and reinforce their strengths and address their weaknesses immediately. This type of precise and direct guidance is not offered by traditional teaching methods, which often rely on delayed feedback. This explains why the quality of the educational digital products produced by the experimental group was higher than that of the control group.

The use of AI-powered smart assistants has contributed to enhancing students’ positive and sustained engagement with digital learning skills by providing an interactive learning environment that encourages participation and experimentation. The continuous interaction facilitated by these AI-powered smart assistants motivated students to exert more effort and focus more intently on producing innovative digital products, unlike the control group, which approached digital learning skills routinely and relied on less stimulating traditional methods.

These AI-powered smart assistants are distinguished by their ability to personalize and guide learning content according to each student’s individual needs. This led to a rapid and effective response in providing educational content tailored to students’ needs, abilities, and interests. This personalization fostered a sense of individual self-interest among the students in the experimental group and enhanced their ability to effectively address digital entrepreneurial challenges compared to the control group.

AI-powered assistants helped students explore multiple alternatives and generate higher-quality, more creative ideas. This was achieved by providing intelligent recommendations based on the analysis of data and information available to the learner. This enhanced students’ ability to think outside the box and create high-quality, innovative digital products compared to the control group, which lacked this diverse range of intelligent support. The students in the experimental group developed advanced digital skills, such as using AI assistants and platforms, analyzing and extracting important information, and using modern technological tools with high proficiency. These advanced skills clearly enabled them to produce higher-quality, innovative digital products than the group that did not use AI assistants.

AI-powered assistants helped reduce the difficulties students typically face, particularly those related to accessing information or the complexity of the tools used. AI provides continuous technical and educational support. This led to a reduction in anxiety and stress levels associated with performing difficult educational skills and thus improved the quality of the final entrepreneurial digital products.

One of the theories underpinning AI-powered smart assistants is “AI-assisted learning,” which emphasizes that AI-powered smart assistants help deliver flexible educational or training content that considers individual student differences. This provides each learner with a personalized experience based on their level and pace of progress, thus contributing to improved learning outcomes.

Digital assistants are also based on AI-enhanced learning, which views artificial intelligence as a cognitive partner that contributes to developing critical thinking and innovation skills through continuous interactive stimulation. This is clearly demonstrated in the quality of the pioneering digital products created by students using AI-powered smart assistants. Furthermore, the digital assistant theory highlights the importance of building AI-powered smart assistants that facilitate student interaction, provide diverse educational incentives, and support them during the development of pioneering digital skills. This helped boost student motivation, which was reflected in increased student participation and improved performance in producing an innovative digital product.

In light of the above, it can be confirmed that designing AI-powered smart assistants using artificial intelligence represents a qualitative leap in modern teaching methods and achieves significant effectiveness in developing the skills of producing innovative digital products among graduate students. This scientifically and logically explains the superiority of the experimental group over the control group. It is also clear that the achieved results are not merely a reflection of the digital aids, but rather an effective application of modern educational theories that have proven their efficacy in smart learning environments.

### 9.3. The Answer to the Third Question, Which Stated: What Is the Impact of AI-Powered Smart Assistants on Developing Future-Oriented Problem-Solving Skills as a Complex Cognitive Skill Among University Students?

To answer this question, the following hypothesis was formulated: There is no statistically significant effect at the 0.05 level of AI-powered smart assistants on the development of future-oriented problem-solving skills, as a complex cognitive skill, among graduate students, as measured by the Future-Oriented Problem-Solving Scale.

To test the validity of this hypothesis, a *t*-test was used for two independent samples to compare the scores on the Future Problem-Solving Scale. Table 4 shows the results of the *t*-test for the significance of the differences between the mean scores of the experimental and control groups on the Future Problem-Solving Scale.

Table 4 shows that the calculated t-value (10.214) is significant at the (0.05) level, with both sides being significant and (80) degrees of freedom. This indicates a statistically significant difference between the experimental and control groups in the post-test of the Future Problem-Solving Scale.

**Figure 3:**
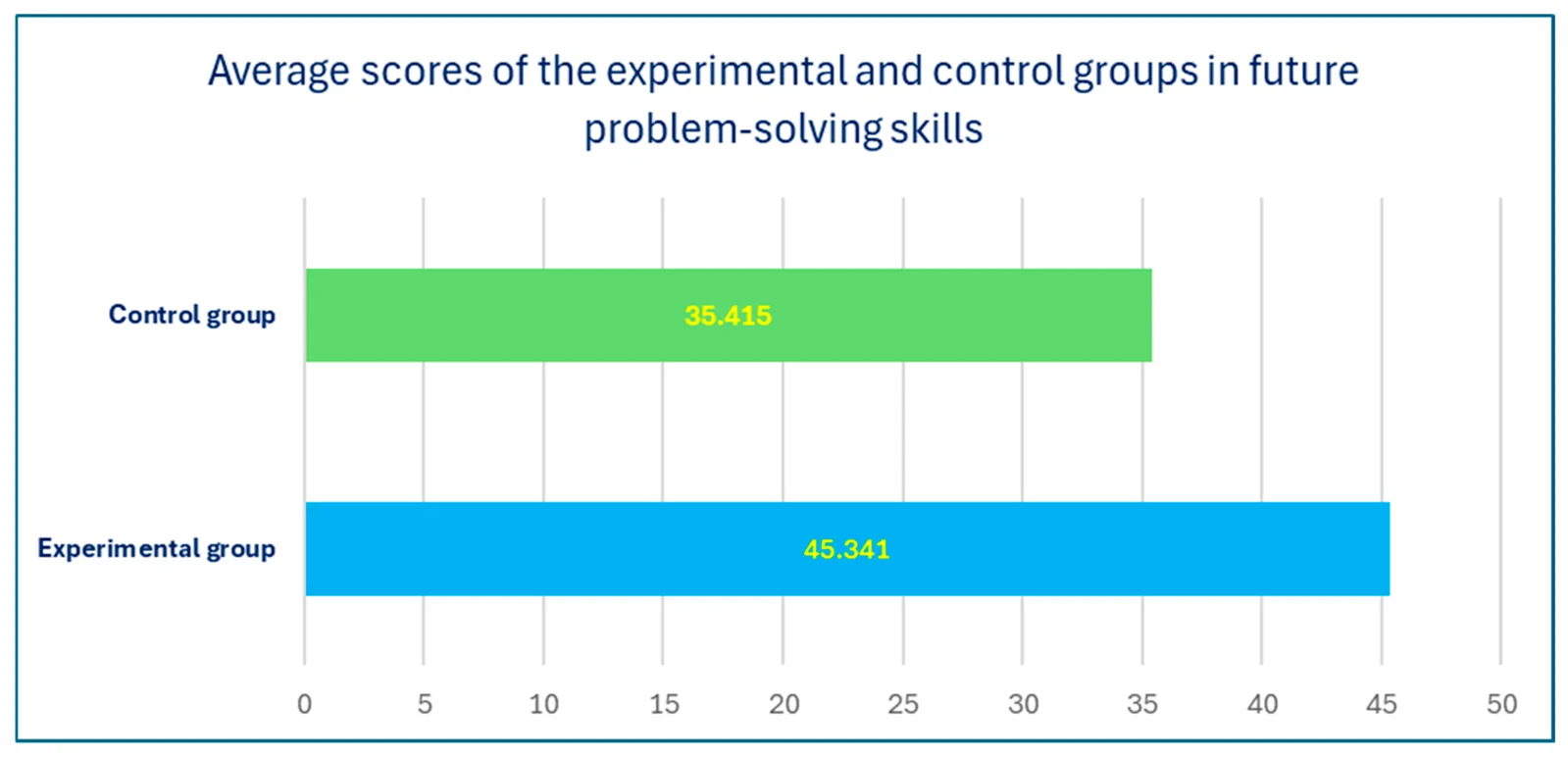
Difference between the experimental and control groups in developing future problem-solving skills.

Therefore, the second research hypothesis was rejected due to a statistically significant difference at the (0.05) level between the mean scores of the experimental and control groups on the post-test of the Futures Problem-Solving Scale for graduate students, in favor of the experimental group that used AI-supported digital tools. Figure 3 illustrates the difference between experimental and control groups in developing future problem-solving skills.

The foregoing suggests that AI-powered smart assistants do not merely play a technical role but rather constitute an effective cognitive medium that enhances student interaction with the learning environment and stimulates critical, analytical, and forward-looking thinking. AI-powered smart assistants present students with unconventional learning situations that require them to process unfamiliar data and consider innovative, future-proof solutions—the very essence of future-oriented problem-solving skills.

Smart assistant platforms are platforms for honing proactive thinking; they are interactive learning environments that combine data analysis, content personalization, and immediate processing of learning situations. This enabled learners to engage in complex simulations, preparing them to tackle unconventional and unexpected problems. Through these platforms, students learned how to anticipate problems, understand their changing patterns, and plan alternative solutions before they occur—a process known as predictive and design-based future thinking.

Smart learning aids are tools for developing cognitive flexibility. Solving future problems requires a high level of mental flexibility and the ability to adapt to new and unexpected data and variables. Smart learning aids contribute to developing this ability by providing dynamic learning scenarios that change with the learner’s input. This leads them to constantly restructure their mental models and develop non-linear problem-solving strategies—a characteristic not readily available in traditional learning environments.

Intelligent Scenarios: One of the most prominent advantages of AI-powered smart assistants is that they do not merely provide information but rather construct learning experiences based on realistic future scenarios. In these scenarios, the learner analyzes the context, prioritizes tasks, and formulates innovative and proactive solutions. This learning style develops contextual reasoning and strategic planning skills, which are essential for solving future problems. AI-powered smart assistants have contributed to shifting education from a process of rote memorization to developing Higher-order cognitive abilities, particularly those related to data analysis, cause-and-effect relationships, and the construction of flexible hypotheses. This explains the significant difference in performance between the experimental group and the control group, who became more capable of handling complex future problems.

AI-powered smart assistants have also helped break down the traditional rigidity of knowledge delivery. Students have moved from being passive recipients to active participants in shaping the learning experience, transforming it into a personalized experience that suits their abilities and learning style. The AI-powered smart assistant, capable of monitoring learner progress, providing immediate feedback, and adapting content based on performance, transforms the process of solving future problems into a dynamic and enriching learning experience, full of challenges and intellectual engagement.

These results are not limited to the educational context but extend to the professional reality for which university students are preparing. The skills enhanced through AI-powered smart assistants, such as problem prediction, proactive thinking, variable analysis, and multi-scenario planning, are all essential skills required by today’s uncertain, rapidly changing, and interdisciplinary job market.

From the above, it is clear that the importance of adopting AI-powered smart assistants as strategic educational tools lies not only in improving academic achievement but also in developing future-oriented thinking skills and achieving a qualitative leap in the quality of university education outcomes, especially at the postgraduate level, which requires specialized preparation to address complex future problems in evolving research or professional contexts.

## 10. Discussion

The current research aimed to develop digital entrepreneurial skills among graduate students by designing AI-powered smart assistants. The research questions were as follows:

### 10.1. What Is the Instructional Design Model for AI-Powered Smart Assistants in an e-Learning Environment, Aimed at Developing Digital Entrepreneurship Skills and Future-Oriented Cognitive Problem-Solving Skills Among University Students?

The first research question sought to develop and validate an instructional design model that integrates AI-powered smart assistants within a structured e-learning environment to support digital entrepreneurship and future-oriented problem-solving among graduate students. The proposed instructional model was developed based on established instructional design principles and reviewed by educational technology and information technology specialists before its implementation in the study.

Instead of functioning as independent learning tools, the AI-powered smart assistants are integrated into a broader educational framework that combines structured learning activities, instructor guidance, continuous feedback, and realistic learning tasks. This educational integration aims to encourage active student participation, guided practice, and reflective interaction throughout the learning process. Accordingly, the proposed model represents an educational approach in which AI-powered smart assistants become one component of a pedagogically designed learning environment, rather than the sole factor influencing learning outcomes.

The development and validation of this instructional model provide practical guidance for integrating AI-powered smart assistants into higher education, ensuring that technological capabilities align with sound pedagogical principles. This approach aligns with recent studies that emphasize that the educational value of generative AI depends on its purposeful integration into instructional design, rather than the technology itself (Daniel et al., 2025; Zhai et al., 2024).

Therefore, the primary research question was addressed by developing an instructional model that integrates AI-powered intelligent assistants within a structured educational framework, suitable for supporting higher-level cognitive learning in higher education. Subsequent sections discuss the effectiveness of this integrated instructional approach in light of measured learning outcomes.

### 10.2. What Is the Impact of AI-Powered Smart Assistants Developing Digital Entrepreneurship Skills Among University Students?

The results showed that students who participated in an educational intervention that integrated AI-powered smart assistants within a structured learning environment achieved significantly higher scores on a digital entrepreneurship product evaluation test compared to the control group. Because the intervention combined AI-powered smart assistants with structured learning activities, instructor guidance, and realistic learning tasks, these results should be interpreted as reflecting the educational value of the integrated approach, rather than solely the contribution of the AI-powered smart assistants.

The large effect size (Cohen’s d = 1.08; η^2^ = 0.51) indicates that the educational intervention was associated with substantial improvements in the quality of students’ digital entrepreneurship products. While the study design does not allow for the isolation of the individual contribution of each educational component, the results suggest that integrating AI-powered smart assistants within a carefully designed educational framework can provide effective support to students as they engage in entrepreneurial learning activities.

These findings are consistent with previous studies (Mohammed & Hassan, 2024; Halaweh, 2023; Anderson & Patel, 2024; Aniella & Gabriel, 2025; Tsakeni et al., 2025), which indicated that integrating AI-powered learning tools into well-designed learning environments enhances student engagement, digital productivity, and entrepreneurial learning. The current findings expand upon this evidence by demonstrating the feasibility of effectively integrating AI-powered intelligent assistants into structured learning environments that encourage students to actively participate in the design, optimization, and evaluation of digital products.

From a cognitive perspective, digital entrepreneurship requires learners to organize knowledge, identify opportunities, generate and evaluate alternative solutions, and make informed design decisions. Therefore, the observed differences between the two groups suggest that the integrated learning environment likely supported these higher-order cognitive processes as students engaged in genuine entrepreneurial learning tasks. However, because these results were achieved through a comprehensive instructional intervention, they should not be attributed solely to AI-powered smart assistants.

Overall, the findings suggest that integrating AI-powered smart assistants within a structured educational framework can support digital entrepreneurship learning in higher education. Future studies using experimental designs capable of isolating the effects of instructional design, teacher facilitation, and AI-powered support will help clarify the relative contribution of each element to student learning outcomes.

### 10.3. What Is the Impact of AI-Powered Smart Assistants on Developing Future-Oriented Problem-Solving Skills as a Complex Cognitive Skill Among University Students?

The results showed that students who participated in an educational intervention that integrated AI-powered smart assistants within a structured learning environment scored significantly higher on the futures-solving scale compared to the control group. Because the intervention combined AI-powered smart assistants with structured learning activities, instructor guidance, and real-world learning tasks, these results should be interpreted as reflecting the contribution of the integrated educational approach, rather than the effect of the AI-powered smart assistants alone.

The large effect size (Cohen’s d = 2.26; η^2^ = 0.57) indicates that participation in the educational intervention was associated with significant improvements in students’ futures-solving performance. While this study cannot isolate the contribution of each educational component, the results suggest that integrating AI-powered smart assistants within a carefully designed educational framework may support learners in analyzing complex situations, evaluating alternative solutions, and engaging in future-oriented thinking.

These findings are consistent with previous research (Hamed, 2023; Mohammed & Hassan, 2024; Alshammari & Khan, 2024; Yang, 2026), which indicates that AI-enabled learning environments, when integrated into structured instructional designs, contribute to increased learner engagement, enhanced reflective thinking, and improved ability to solve complex problems.

Student reflections collected at the end of the program further support this interpretation. Many participants reported that the AI-enabled learning environment helped them organize their digital projects more effectively, generate alternative ideas, and approach complex problems more systematically. These perceptions provide additional qualitative support for the quantitative findings, keeping in mind that they are learners’ personal experiences and not direct evidence of causation.

From a cognitive perspective, future-oriented problem-solving requires learners to analyze changing situations, anticipate potential outcomes, compare alternative courses of action, and make informed decisions under conditions of uncertainty. The significant differences between the two groups suggest that the integrated learning environment provided students with greater opportunities to engage in higher-order thinking processes during learning activities. However, these results should be interpreted within the context of holistic instructional design, rather than attributing them solely to AI-powered smart assistants. Overall, the findings suggest that integrating AI-powered smart assistants within a structured educational framework may support the development of future problem-solving skills in higher education. However, further research using experimental designs capable of isolating the effects of AI-powered support, instructional design, and teacher facilitation is needed to better understand the relative contribution of each element to students’ cognitive development.

Beyond the direct educational implications, the results also highlight broader implications for the sustainable integration of AI in higher education. When integrated into pedagogically sound teaching practices, AI-powered smart assistants may contribute to more efficient and flexible learning environments. At the same time, higher education institutions should promote the responsible and sustainable use of artificial intelligence by balancing their educational benefits with the ethical, technical and environmental considerations associated with AI technologies.

The findings suggest that AI-powered smart assistants can represent educational innovations that foster digital entrepreneurship and future-oriented problem-solving, supporting Sustainable Development Goal 9 by integrating advanced digital technologies into higher education. While improved educational practices may indirectly contribute to SDG 4 by enhancing the quality of learning, this study does not specifically address the issue of equal opportunities or inclusive education. Therefore, the implications of these findings are primarily interpreted within the context of innovation, digital transformation, and technology-driven educational development.

## 11. Research Limitations

The current research was limited to the following parameters:

1- Subject Matter: The subject matter focused on the practical component of the “Digital Platforms” course.
2- The following entrepreneurial digital skills:
  ▪ Designing an intelligent assistant for creating interactive electronic content
  ▪ Designing a chatbot
  ▪ Designing an intelligent platform
  ▪ Designing a system for analyzing educational data
  ▪ Designing a virtual simulation to solve an educational problem
3- Future problem-solving skills: anticipation, prediction, planning, and visualization
4- Human scope: Graduate students at the College of Education at a higher education institution
5- Time scope: The first semester of the academic year 2024/2025
6- Spatial scope: The research was conducted at the College of Education at a higher education institution

Despite its contributions, this study has several limitations. Its findings are based on a single higher education institution and a limited sample of graduate students, which may restrict their generalizability. Furthermore, the quasi-experimental design and focus on short-term outcomes may not fully reflect the long-term effects of AI-powered smart assistants. Therefore, future research should replicate this study with more diverse samples and longitudinal designs to enhance the generalizability and sustainability of the findings.

## 12. Conclusions

Advances in artificial intelligence (AI) offer new opportunities to enhance teaching and learning in higher education when integrated into pedagogically sound learning environments. This study examined a quasi-experimental learning intervention that integrated AI-powered intelligent assistants into a structured e-learning environment designed to support the development of digital entrepreneurship and future-oriented problem-solving skills among graduate students. The results showed that students participating in the learning intervention achieved significantly higher levels of digital entrepreneurship and future-oriented problem-solving skills compared to the control group. Because the intervention combined AI-powered intelligent assistants with structured learning activities, instructor guidance, and real-world learning tasks, these results should be interpreted as reflecting the educational value of an integrated approach, rather than the effect of AI-powered intelligent assistants alone.

From a cognitive perspective, the findings suggest that integrating AI-powered intelligent assistants within a carefully designed learning framework can effectively support learning activities that require knowledge organization, analytical thinking, creative idea generation, evaluation of alternative solutions, strategic planning, and evidence-based decision-making. These cognitive processes are closely linked to digital entrepreneurship and future-oriented problem-solving and represent important dimensions of higher-order cognitive skills in higher education. This study contributes to the growing literature on AI-assisted learning by providing empirical evidence that structured learning interventions incorporating AI-powered intelligent assistants can support learning outcomes related to digital entrepreneurship and future-oriented problem-solving. Rather than viewing AI-powered intelligent assistants as independent agents in determining learning outcomes, the findings highlight their potential educational value when integrated with appropriate instructional design and pedagogical support.

These findings also have important implications for educational practice. Higher education institutions may benefit from integrating AI-powered intelligent assistants into well-designed learning environments that foster active learning, reflection, collaboration, and real-world problem-solving, rather than primarily using AI as an information retrieval tool. This integration would better prepare students for the demands of digital work environments while supporting the development of higher-order cognitive skills.

The findings suggest that higher education institutions and policymakers should consider supporting the appropriate pedagogical integration of AI-enabled smart assistants within institutional teaching and learning strategies. This includes investing in faculty professional development, encouraging the responsible and ethical use of generative AI, and establishing institutional guidelines that promote effective instructional design, ensuring that AI complements, rather than replaces, meaningful teaching and student engagement.

Overall, the findings highlight the potential of AI-powered smart assistants to support innovation-oriented higher education by enhancing higher-order cognitive skills, digital entrepreneurship, and future-oriented problem-solving. These results are fundamentally aligned with Sustainable Development Goal 9, which focuses on innovation and technological advancement, while indirectly contributing to improved education quality under Sustainable Development Goal 4 through enhanced pedagogical practices rather than inclusive education initiatives.

Finally, this study addresses a significant gap in the literature by examining digital entrepreneurship and future-oriented problem-solving within a single educational intervention. However, because the intervention comprises multiple integrated educational components, future research should employ experimental designs capable of isolating the individual contributions of AI-enabled smart assistants, instructional design, and teacher facilitation. Such research would provide a clearer understanding of the mechanisms by which AI-enabled learning environments influence higher-level cognitive development in higher education.

## Figures and Tables

**Table 1:** Results of the *t*-test for the Product Evaluation Card and the Future Problem-Solving Scale in the pretest.

Instrument	Group	*N*	Mean	SD	D.F	t-Value	Significance Level	Significance
Product Evaluation Card	Experimental	41	5.12	1.25	80	1.11	0.271	Not Significant
	Control	41	4.78	1.53				
Future Problem-Solving Scale	Experimental	41	20.37	1.47	80	1.60	0.114	Not Significant
	Control	41	19.83	1.26				

**Table 2:** Pearson correlation coefficient matrix between scale dimensions and overall score.

Dimension	Visualization	Prediction	Expectation	Planning
Visualization	1			
Prediction	0.526 *	1		
Expectation	0.389	0.351	1	
Planning	0.209	0.173	0.594 *	1
Total Scale	0.743 **	0.663 **	0.794 **	0.669 **

* *p* < 0.05; ** *p* < 0.01.

**Table 3:** The value of “t” and its statistical significance between the mean scores of the experimental and control groups on the digital entrepreneurial product evaluation form.

Group	Instrument	N	Mean	SD	D.F	t-Value	Significance	Cohen’s d	Effect Size
Experimental	Entrepreneurial Digital Product Evaluation Card	41	8.561	1.097	80	9.188 **	Significant	1.08	0.51
Control		41	6.366	1.067					

** *p* < 0.01.

**Table 4:** t-value and its statistical significance between the mean scores of the experimental and control groups on the Future Problem-Solving Scale.

Group	Instrument	*N*	Mean	SD	D.F	t-Value	Significance	Cohen’s d	Effect Size
Experimental	Future Problem-Solving Scale	41	45.341	4.531	80	10.214 **	Significant	2.26	0.57
Control		41	35.415	4.266					

** *p* < 0.01.

## Data Availability

The data supporting the findings of this study are available from the corresponding author upon reasonable request.
